# Serum exosomal miR-192 serves as a potential detective biomarker for early pregnancy screening in sows

**DOI:** 10.5713/ab.22.0422

**Published:** 2023-05-04

**Authors:** Ruonan Gao, Qingchun Li, Meiyu Qiu, Su Xie, Xiaomei Sun, Tao Huang

**Affiliations:** 1 College of Animal Science and Technology, Shihezi University, 221 North Fourth Road, Shihezi 832000, China; 2 Institute of Biotechnology, Xinjiang Academy of Animal Science, Urumqi, 830000, China; 3 Key Laboratory of Animal Breeding and Reproduction of Minstry of Education, College of Animal Science and Technology, Huazhong Agricultural University, Wuhan, 430070, China; 4 Xinjiang Pig Breeding Engineering Technology Research Center, Xinjiang Tecon Husbandry S&T Co. Ltd, Changji, 831100, China

**Keywords:** Biomarkers, Early Pregnancy, Exosomal miRNAs, ITGA4, miR-192

## Abstract

**Objective:**

The study was conducted to screen differentially expressed miRNAs in sows at early pregnancy by high-throughput sequencing and explore its mechanism of action on embryo implantation.

**Methods:**

The blood serum of pregnant and non-pregnant Landrace×Yorkshire sows were collected 14 days after artificial insemination, and exosomal miRNAs were purified for high throughput miRNA sequencing. The expression patterns of 10 differentially expressed (DE) miRNAs were validated by quantitative reverse transcription-polymerase chain reaction (qRT-PCR). The qRT-PCR quantified the abundance of serum exosomal miR-192 in pregnant and control sows, and the diagnostic power was assessed by receiver operating characteristic (ROC) analysis. The target genes of DE miRNAs were predicted with bioinformatics software, and the functional and pathway enrichment analysis was performed on gene ontology and the Kyoto encyclopedia of genes and genomes terms. Furthermore, a luciferase reporter system was used to identify the target relation between miR-192 and integrin alpha 4 (ITGA4), a gene influencing embryo implantation in pigs. Finally, the expression levels of miRNAs and the target gene ITGA4 were analyzed by qRT-PCR, and western blot, with the proliferation of BeWo cells detected by cell counting kit-8 (CCK-8).

**Results:**

A total of 221 known miRNAs were detected in the libraries of the pregnant and non-pregnant sows, of which 55 were up-regulated and 67 were down-regulated in the pregnant individuals compared with the non-pregnant controls. From these, the expression patterns of 10 DE miRNAs were validated. The qRT-PCR analysis further confirmed a significantly higher expression of miR-192 in the serum exosomes extracted from pregnant sows, when compared to controls. The ROC analysis revealed that miR-192 provided excellent diagnostic accuracy for pregnancy (area under the ROC curve [AUC] = 0.843; p>0.001). The dual-luciferase reporter assay indicated that miR-192 directly targeted ITGA4. The protein expression of ITGA4 was reduced in cells that overexpressed miR-192. Overexpression of miR-192 resulted in the decreased proliferation of BeWo cells and regulated the expression of cell cycle-related genes.

**Conclusion:**

Serum exosomal miR-192 could serve as a potential biomarker for early pregnancy in pigs. miR-192 targeted *ITGA4* gene directly, and miR-192 can regulate cellular proliferation.

## INTRODUCTION

Exosomes are cell-derived vesicles present in all eukaryotic fluids, including blood, urine, and cell culture media [1]. First discovered in 1986, exosomes in mammalian reticulocytes range in diameter from 30 to 100 nm [2]. Exosomes contain a variety of cellular molecular components including proteins, RNA, lipids, and metabolites. Exosomes in body fluids are exceedingly stable, resulting in specialized biological functions that are crucial for molecular coagulation, intercellular molecular conduction, and internal waste management. Therefore, increasing attention has been directed toward the application of exosomes in clinical practice, e.g. as biomarkers of diseases and physiological processes [3].

microRNAs (miRNAs) are a class of endogenous non-coding small RNA molecules with a length of 18 to 25 nt. miRNAs specifically bind to partially or completely complementary bases in the 3′-UTR region of its target gene mRNA, inducing mRNA degradation or inhibiting protein translation. miRNAs are involved in numerous biological processes including early development, cell proliferation, differentiation, apoptosis, cellular death, fat metabolism, and cancer development [4–7].

miRNAs are intimately involved in reproduction, regulating trophoblast invasion, endometrial receptivity, and maternal-fetal interactions during embryo implantation [8]. The miRNAs let-7a and let-7b can directly bind to the 3-UTR region of mucin 1 and participate in the process of embryo implantation [9]. Human chorionic gonadotropin (HCG) down-regulates olfactomedin1 (OLFM1) by stimulating the expression of miR-212 and the C-terminal binding protein 1 (CTBP1) in the fallopian tubes and endometrial epithelial cells, thereby inducing the development of the endometrium to the receptive stage for embryo adhesion [10]. During the implantation period of patients with repeated embryo implantation failure (RIF), the significant up-regulation of miR-148a-3p in endometrial tissue may result from the down-regulation of the homeobox C8 (*HOXC8*) gene. The subsequent inhibition of differentiation in endometrial stromal cells hinders the process of decidualization and can lead to embryo implantation failure [11].

In addition, expression of miR-200c, miR-17, or miR-192 in untransformed human colon fibroblasts can downregulate 85% of all predicted target genes [12]. Overexpression of miR-192 can downregulate matrix metallopeptidase 11 (MMP-11) expression, promote apoptosis, and reduce the proliferation, migration, and invasion of osteosarcoma (OS) cells [13].

miR-192 may play an important role in mediating disease occurrence, diagnosis, and treatment, but whether it is involved in early embryo implantation in sows is unclear. Currently, there is no reporting of exosome miRNAs in the serum of sows during early pregnancy. Therefore, by studying the normal expression of exosomal miRNA in sow serum during early pregnancy, we assessed the potential association between miR-192 and implantation-related gene integrin alpha 4 (ITGA4) expression in sows serum and developed potential diagnostic biomarkers to detect sows early in pregnancy.

## MATERIALS AND METHODS

### Sample processing

Ten multiparous Landrace-Yorkshire Hybrid sows, aged 17 to 19 months and weighing 210±10 kg, were inseminated artificially when estrus signs were observed. After 14 days, peripheral blood samples were collected using BD vacutainer SST II Advance collection tubes (Belliver Industrial Estate, Plymouth, PL67BP, UK) and stored in −80°C. After 35 days of artificial insemination, the pregnancy of the animals was detected by B-ultrasonography. All procedures involving animals were approved by the Animal Care Committee of Shihezi University, China (A2019-144-01).

### RNA isolation and Illumina sequencing

ExoRNeasy serum/plasma Midi kit (#77144; Exiqon QIAGEN, Dusseldorf, Germany) was used to extract serum exosomal miRNA. Briefly, 1 mL serum sample was mixed with an equal volume of XBP buffer, loaded onto an exoEasy spin column, and centrifuged at 5,000×g for 1 min. The solution was then mixed with 3.5 mL XWP buffer, before being washed at 5,000×g for 5 min, and exosomes eluted. miRNA was then extracted and eluted with 14 μL of RNase-free water.

RNA purity and concentration were assessed using a Nano Drop 2000 spectrophotometer (Thermo Scientific, Waltham, MA, USA). Samples with 260/280 ratios above 1.8, and 260/230 ratios above 2.0 were considered acceptable and stored at −80°C. The integrity and concentration of the total RNA were measured with the RNA Nano 6000 Assay Kit for the Agilent Bioanalyzer 2100 system (Agilent Technologies, CA, USA), and RNA libraries of pregnant sample and non-pregnant sample were constructed according to the standard Illumina protocols. Libraries were Solexa-sequenced on an Illumina Genome Analyzer (Beijing Compass Biotechnology Co. Ltd, Beijing, China).

### Alignment and annotation of small RNAs

The original image data files of the high-throughput sequencing were converted to sequenced reads with base calling analysis. Raw FastQ files were processed through custom Perl and python scripts. Clean reads were obtained by removing reads containing ploy-N, with 5′ adapter contaminants; reads without 3′ adapter or the insert tag, containing ploy A or T or G or C; and reads of low quality. Simultaneously, Q20, Q30, and GC-content of the raw data were calculated. A certain range of lengths from clean reads were chosen for all downstream analyses. The small RNA tags were mapped to a reference sequence in Bowtie [14,15] without allowing mismatches, to analyze their expression and distribution on the reference. Mapped small RNA tags were used to identify known miRNAs. miRBase22.0 was used as a reference, while modified software miRDeep2 [16] was used to obtain the identity of the potential miRNA and construct its secondary structures. Custom scripts were used to obtain miRNA counts, as well as base biases on the first position of the identified miRNAs of a certain length, and on each position of all identified miRNAs. The characteristic hairpin structure of the miRNA precursor can be used to predict novel miRNAs. The software miREvo [17] and miRDeep2 [18] were integrated to predict novel miRNAs through the exploration of secondary structures, the Dicer cleavage site, and the minimum free energy of the small RNA tags unannotated in the former steps. To ensure that every unique small RNA was mapped to only one annotation, we followed the following priority rule: known miRNA > rRNA > tRNA > snRNA > snoRNA > repeat > gene > NAT-siRNA > gene > novel miRNA > ta-siRNA. The total rRNA proportion was used as a marker of sample quality [19].

### Prediction of target genes, and analysis of GO and KEGG enrichment

Prediction of the target genes of miRNAs was performed with the miRanda database for animals. Gene ontology (GO) enrichment analysis was used on the target gene candidates of differentially expressed miRNAs (“target gene candidates”). GOseq based Wallenius non-central hypergeometric distribution, which adjusted for gene length bias, was implemented for GO enrichment analysis. Kyoto encyclopedia of genes and genomes (KEGG) ( http://www.genome.jp/kegg/) enrichment analysis was also conducted [20]. KOBAS software was utilized to test for statistical enrichment of the target gene candidates in identified KEGG pathways [21].

### Screening of differentially expressed miRNAs

miRNA expression levels were estimated by transcript per million (TPM) through the following criteria: Normalization formula: Normalized expression = mapped read count/Total reads×1,000,000. Differential expression analysis of two conditions/groups was performed with the DESeq R package (1.8.3). p-values were adjusted using the Benjamini & Hochberg correction method, with a corrected p-value of 0.05 set as the threshold for significantly differential expression.

### Verification of differentially expressed miRNAs by qRT-PCR

To verify the accuracy of miRNA sequencing data, 10 differentially expressed miRNAs were randomly selected for quantitative validation. The downstream primers of these miRNA were sourced from self-priming kits (Table 1), and the upstream primers (Table 1) were designed by TIANGEN (Beijing, China), while U6 was used as a reference gene. Reverse transcription of cDNA was conducted using the miRcute Plus miRNA First-Strand cDNA Synthesis Kit (TIANGEN, China). Tubes were incubated at 42°C for 60 min followed by 3 min at 95°C, then cooled to 4°C where they were held until use. The miRcute Plus miRNA qPCR Detection Kit (TIANGEN, China) was used for quantitative PCR (qPCR). Thermal cycling on a Light Cycler 96 PCR system (Roche, Switzerland), consisted of an initial incubation step at 95°C for 5min, followed by 45 cycles at 95°C for 10 s, 56°C for 20 s and 72°C for 10 s. Data were normalized by ΔCt = Ct(target) − Ct(mean of sample).

### Receiver operating characteristic curve evaluation

We examined the exosomal expression levels of miR-192 in serum samples of 21 pregnant sows and 15 non-pregnant sows by quantitative reverse transcription-polymerase chain reaction (qRT-PCR), using U6 as the internal reference gene. To determine whether the level of exosomal miR-192 could be used to distinguish pregnant sows from nonpregnant ones, we constructed a receiver operating characteristic (ROC) curve to evaluate the discrimination power of miR-192 for pregnancy.

### Prediction and preliminary validation of target gene of miR-192

miRanda [22] ( http://miranda.org.uk/), RNAhybrid [23] (http://bibiserv.techfak.uni-bielefeld.de/rnahybrid), and TargetScan [24] ( http://www.targetscan.org ) were used to predict the target genes ofmiR-192, and to analyze the binding site of miR-192 to porcine *ITGA4* gene.

### Construction of plasmid vector with 3′-UTR of ITGA4

According to the predicted 3′-UTR base recognition site of miR-192 on the target gene, 3′-UTR-ITGA4-W and 3′-UTR-ITGA4-M vector primers were designed (Table 2). This allowed for the 3′-UTR region sequence of the candidate target gene to be amplified. After the PCR products were separated by 1.5% agarose gel electrophoresis, the target bands were cut and purified. The purified PCR product and the psiCHECK-2 vector were both digested with *Xho* I and *Not* I, and the target band was separated by 2% gel electrophoresis. The psiCHECK-2 and target gene fragments were ligated by T4 DNA ligase.

### Cell culture and transfection

PK-15 and BeWo cells were cultured in a medium with 10% fetal bovine serum, and 100 U/mL penicillin DMEM (GIBCO, Carlsbad, CA, USA). Cells were seeded in 12 well plates and cultured in 5% CO_2_ at 37°C for 18 to 24 h until 70% to 80% confluence. The cells were starved with Opti-MEM medium for 2 hours and transfected with Lipofectamine 2000 transfection reagent (Life Technologies, Carlsbad, CA, USA). Each group of samples was set up in three parallel experimental groups with three replicates. The miR-192 mimics, mimics NC (GenanBio Co, Guangzhou, China), ITGA4-W plasmid, and ITGA4-M plasmid were co-transfected, respectively (4 experimental groups were set up: miR-192 + ITGA4-W; miR-192 + ITGA4-M; mimics NC + ITGA4-W; mimics NC + ITGA4-M).

### Luciferase assay

Six hours after transfection, the culture medium was replaced, and 24 hours after transfection the activity of Luciferase and Renilla were measured using a Dual-Luciferase Reporter Assay Kit (Promega Corporation, Madison, WI, USA).

### qRT-PCR detection of target genes

The expression levels of ITGA4 were determined using SYBR Green Real-Time PCR.

All data were analyzed using the formula 2 ΔΔCt [19,25]. The specific primers were synthesized by Sangon Biotech (Shanghai, China) Co., Ltd., and glyceraldehyde-3-phosphate dehydrogenase (GAPDH) was used as a reference gene for normalization (Table 3).

### Western blot analysis

The protein lysate was added to PK-15 cells. After incubation on ice for 30 min, the protein supernatant was extracted by centrifugation, and the protein concentration was detected by BCA method. Western blot analysis was performed according to the standard protocol. ITGA4 and β-actin antibodies were purchased from Cell Signaling Technology (Boston, MA, USA). Chemiluminescent signals were detected using a luminous value by a near-infrared system (Bioworld Technology, St. Louis Park, MN, USA).

### CCK-8 assay

In a 96-well plate, each well was seeded with 1×10^3^ BeWo cells and accepted different treatments. Then, 10 μL enhanced cell counting kit-8 (CCK-8) reagent cell was added to each well 6 hours after transfection, and the absorbance was read at 450 nm.

### Statistical analysis

Statistical analysis was performed using SPSS software version 20.0 (SPSS, Chicago, IL, USA). Data are expressed as the mean and standard error of mean of at least three independent experiments. A Student’s t-test was used for comparison between two groups, while Duncan’s multiple comparison method in one-way analysis of variance was used for comparison among multiple groups. Classification variables are represented by numbers, and differences are compared by the chi-square test. A ROC curve was established to evaluate the cut-off value of exosomal miR-192.

## RESULTS

### Small RNA sequencing analysis

**Figure 1:**
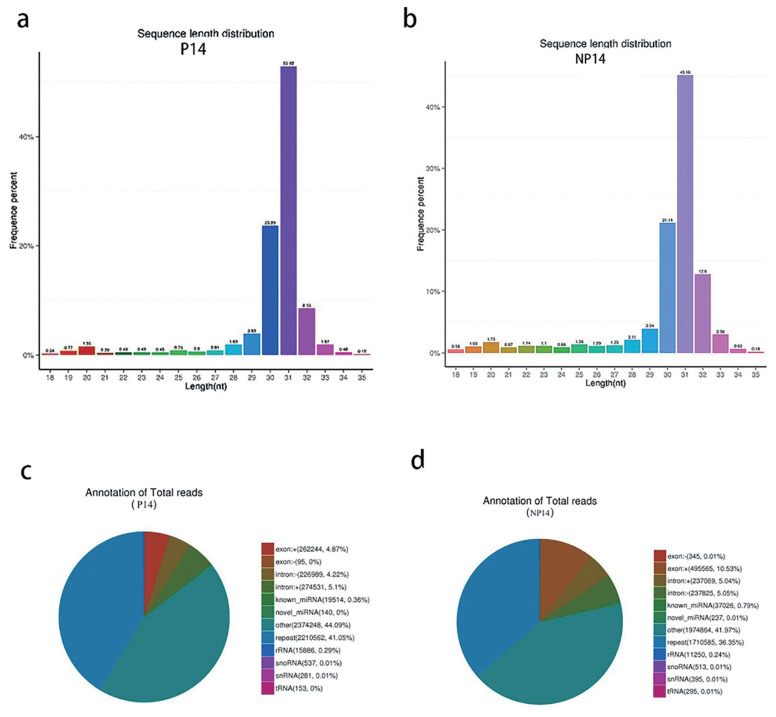
(a) Small RNA fragment length distribution for pregnant sows. (b) Small RNA fragment length distribution for non-pregnant sows. (c) Small RNA classification annotation for pregnant sows. (d) Small RNA classification annotation for non-pregnant sows.

A total of 6,453,029 raw reads were obtained from the library of pregnant sows, and 6,024,905 were obtained from the non-pregnant control group. In general, the length of the animals’ small RNA ranged from 18 to 35 nt. The main peaks of the two libraries were mainly distributed around 31 nt, and the length of miRNAs was generally around 22 nt (Figure 1a, 1b). This variability may be due to different sequencing species or samples [26,27].

The reference sequence alignment analysis revealed that 85.54% and 82.40% of the RNAs in P14 and NP14 groups were matched to the reference sequence, respectively. The total amount of rRNA in the classification annotation results can be used as a quality control standard for a sample. In general, the proportion of total rRNA in good quality animal samples should be less than 40%, with our results (0.24% and 0.29%), indicating very high quality (Figure 1c, 1d).

### Screening of differentially expressed miRNAs

**Figure 2:**
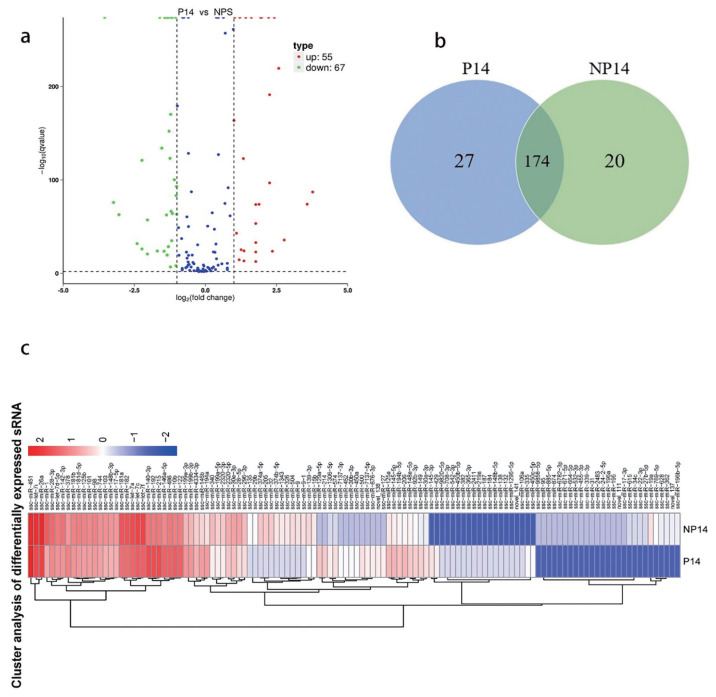
Differential expression of miRNAs between pregnant and non-pregnant sows. (a) Each dot represents an individual miRNA. Blue circles represent no significant difference, while red and green circles indicate significant up-regulation and down-regulation of differential miRNAs, respectively. (b) Differential expression miRNA Venn diagram includes the total number of miRNAs in each circle. Each overlap represents the number of miRNA expressions in the corresponding combinations. (c) The hierarchical clustering analysis of miRNAs clusters log10(TPM+1) values of altered expression (p<0.05, fold change >2). Red strip, high relative expression; blue strip, low relative expression; white strip, no change in gene expression. Color intensity reflects the degree of expression increase or decrease.

Our reads were normalized using trimmed mean of M-values, followed by DEGseq for differential expression analysis. The results revealed that a total of 221 known miRNAs were detected in all samples, of which 55 were found up-regulated and 67 down-regulated in the pregnant group when compared with the control group (Figure 2a, 2b). The TPM of each comparative set of differentially expressed miRNAs from each sample was used for hierarchical cluster analysis, K-means cluster analysis, and SOM (Figure 2c). These results indicate that the expression of miR-451 was the highest in both groups, and miR-196b-5p was the lowest in both groups.

### Verification of differential gene expression

**Figure 3:**
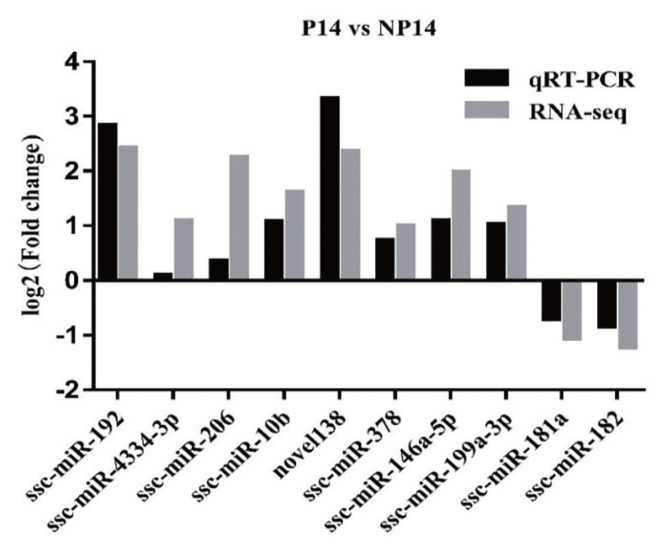
Validation of miRNAs expression by quantitative reverse transcription-polymerase chain reaction.

Ten miRNAs screened differentially expressed (eight up-regulated and two down-regulated) in front were randomly selected for qRT-PCR verification. The results showed that the miRNA expression trends were consistent with that of high-throughput sequencing (Figure 3), miR-192 was confirmed to be upregulated by 5.77-fold in pregnant sows versus nonpregnant control sows (p<0.05).

### Prediction of target genes and bioinformatics analysis

A total of 2,376 target genes were predicted for the nine miRNAs (ssc-miR-192, ssc-miR-4334-3p, ssc-miR-206, ssc-miR-10b, novel-138, ssc-miR-378, ssc-miR-146a-5p, ssc-miR-182, ssc-miR-181a. Many of the predicted genes were connected to reproduction, such as ssc-miR-192 and its’ target genes integrin alpha 4 (*ITGA4*), integrin Beta 6 (*ITGB6*), mitogenactivated protein kinase kinase kinase 1 (*MAP3K1*); ssc-miR-146a-5p and its’ target genes, such as SMAD family member 3 (*Smad3*), ras homolog family member A (*RHOA*), and SMAD family member 4 (*Smad4*), nuclear receptor coactivator 3 (*NCOA3*); ssc-miR-378 and its’ target genes,such asrecombinant ribosomal protein S6 kinase beta 2 (*RPS6KB2*), mitogen-activated protein kinase 1 (*MAPK1*), SMAD family member 3 (*Smad5*), bone morphogenetic protein 6 (*BMP6*); and ssc-miR-181a with its’ target gene integrin beta 1 (*ITGB1*).

**Figure 4:**
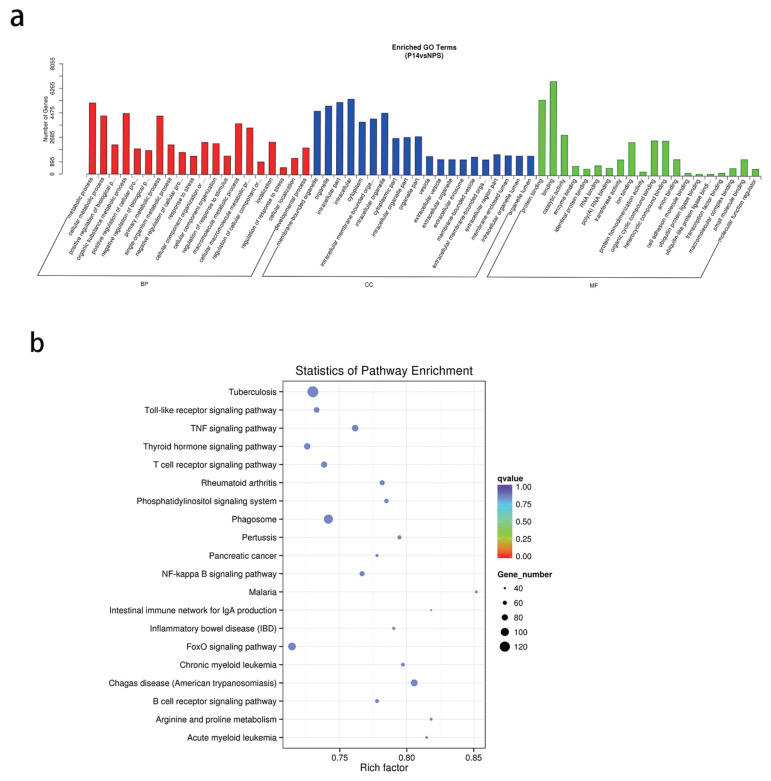
(a) Candidate target gene GO enrichment histogram. (b) Signaling pathway analysis of target genes of deregulated miRNAs by KEGG enrichment. GO, gene ontology; KEEG, Kyoto encyclopedia of genes and genomes.

Gene ontology (GO) analysis results (Figure 4a) presented a total of 8,950 target genes that were significantly enriched in function. 1,965 of these were significantly enriched in developmental processes, 1,371 genes in vesicles, 1,143 genes in extracellular vesicles, and 1,135 genes in extracellular exosomes. These target genes may play an important role in the regulation of pregnancy.

Kyoto encyclopedia of genes and genomes (KEGG) pathway analysis showed that the target genes involved 275 signal pathways, including tumor necrosis factor (TNF), forkhead box O (FoxO), Notch, transforming growth factor-β (TGF-β), mechanistic target of rapamycin signaling pathways (Table 4; Figure 4b). The study of the signaling transduction pathways associated with these genes could help us to further understand the mechanisms of regulating pregnancy.

### Diagnostic implication of serum exosomal miR-192

**Figure 5:**
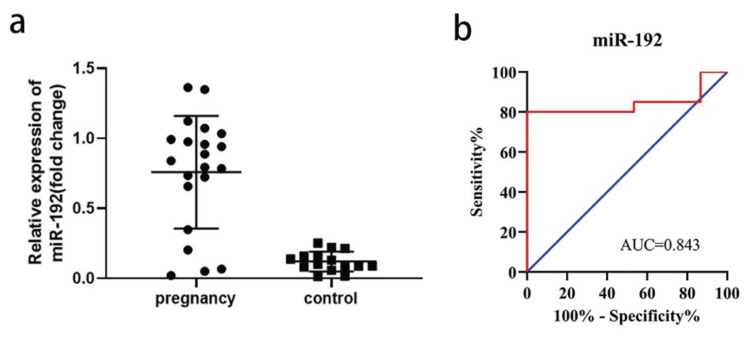
(a) The expression levels of miR-192 in serum detected in pregnant and non-pregnant control sows. (b) ROC analysis for distinguishing pregnancy group from controls using serum exosomal miR-192. ROC, receiver operating characteristic.

The expression amount of serum exosomal miR-192 in the pregnant group was 5.77 times that in the control (p<0.01) (Figure 5a). The ROC analysis showed that serum exosomal miR-192 was a reliable biomarker for differentiating pregnant samples from non-pregnant controls. The AUC of miR-192 was 0.843 (95% CI, 0.727 to 1.000; p = 0.0002), while the sensitivity and specificity were 81.0% and 100% respectively (Figure 5b). These results demonstrate that the exosomal miR-192 in serum has significant potential as a diagnostic biomarker to detect early pregnancy in sows.

### Identification of ITAG4 as a target gene of miR-192

**Figure 6:**
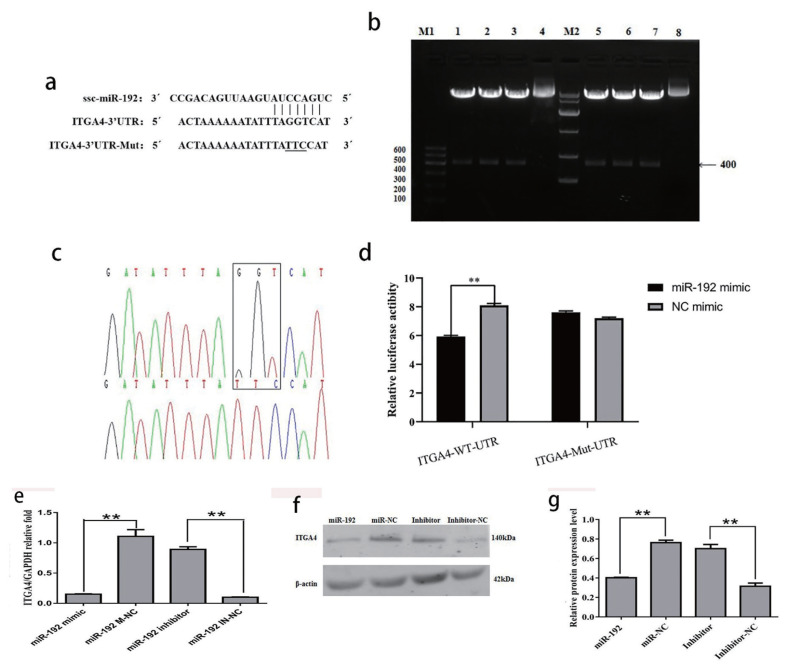
(a) Verification of downstream target genes of miR-192. The 3′-UTR fragment of ITGA4 in sows containing the binding sites of miR-192. (b–c) the electrophoresis results of the digested ITGA4 gene 3′-UTR psichecktm-2 vector. lane M1, DNA Maker DL 600; lane 1–3, the double enzyme digestion of ITGA4-W recombinant plasmid; lane 4, the ITGA4-W recombinant plasmid; lane 5–7, the double enzyme digestion of ITGA4-M recombinant plasmid; lane 8, the ITGA4-M recombinant plasmid; M2, DNA Maker DL 10000. (d) Luciferase reporter assays. PK-15 cell was co-transfected with reporters containing the wild-type or mutant form of 3′-UTR of PER1 mRNA and control miRNA or miR-192 mimic. (e) mRNA expression of ITGA4 in PK-15 cells after the transfection of miR-192. (f-g) Expression of ITGA4 protein in PK-15cell lines under treatment of miR-192mimics or NC sequence. Data are presented as the mean±standard error of the mean, ** p<0.01.

ITGA4 was screened as the target gene of ssc-miR-192. The binding site of ssc-miR-192 was taggtca (Figure 6a). The recombinant psiCHECK- 2 plasmids with wild-type and mutation 3′- UTR of ITGA4 were constructed using primer site-directed mutation of miRNA-binding sites. The ITGA4-WT and ITGA4-MT plasmids were successfully constructed (Figure 6b, 6c). In the cells transfected with wild-type luciferase vector, the luciferase activity of the miR-192 mimic group was significantly decreased by 27.0% (p<0.01). However, there was no significant difference between the groups of cells transfected with the mutant luciferase vector (p>0.05) (Figure 6d). The mRNA expression of ITGA4 in the miR-192 mimic group was significantly lower than that of the negative control group (p<0.01), and the mRNA expression of ITGA4 in the miR-192 inhibitor group was significantly higher than that of the negative control group (p<0.01; Figure 6e). The protein expression level of ITGA4 transfected with miR-192 mimic was significantly lower than that of its negative control group (p<0.01), and the amount of ITGA4 protein in the miR-192 inhibitor group was significantly higher than that of its negative control group (p<0.01). miR-192 exhibited inhibitory effects on mRNA and protein expression of ITGA4 (Figure 6f, 6g).

### Regulation of proliferation of BeWo cells by miR-192

**Figure 7:**
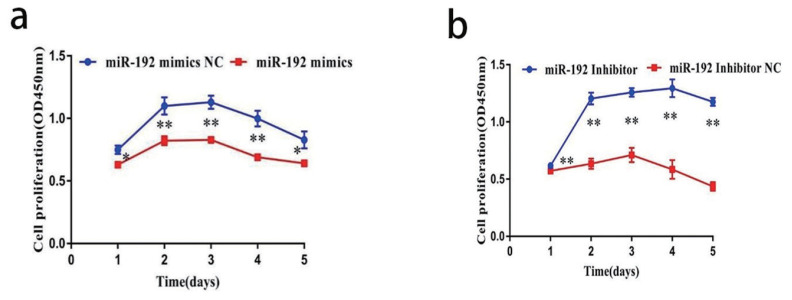
(a, b) CCK-8 assay was used to evaluate the effect of BeWo cells. Data are presented as the mean±standard error of the mean. * p<0.05, ** p<0.01. CCK-8, cell counting kit-8.

The proliferation of cells in the miR-192 mimic group was significantly lower than that in the miR-192 mimic NC group (p<0.01). However, the proliferation of the miR-192 inhibitor group at 96 hours after transfection was significantly higher than that of the miR-192 inhibitor NC. The results indicated that miR-192 over-expression can inhibit the proliferation of BeWo cells, while inhibition can increase the cell’s proliferation capacity (Figure 7a, 7b).

## DISCUSSION

The traditional methods of diagnosing a sow’s pregnancy status, such as hormone measurement, detection of prostaglandin in blood, and estrogen concentration in urine and feces [18,25–28] can result in identification errors (“false oestrus”), while ultrasound diagnosis requires expensive equipment and the experience of the operator can influence the results [30]. Exosomes, as a form of transport of extracellular miRNAs, can protect miRNAs from degradation by ribonuclease, making exosomal miRNAs highly stable [31]. Exosomal miRNAs have unique roles in mediating intercellular communication. The unique stability and spatiotemporal specificity in body fluids make it an ideal novel biomarker. By analyzing the differentially expressed exosomal miRNAs in pregnant and non-pregnant sows’ serum and their regulatory mechanisms, we identified that these differentially expressed miRNAs can be used to detect sows in early pregnancy. The new diagnostic biomarkers can also provide a reliable theoretical basis for the early diagnosis of sows.

Many miRNAs are associated with trophoblast differentiation, proliferation, invasion and migration, and pregnancy disorders such as preeclampsia, intrauterine growth restriction, or placenta accrete [32–35]. miRNAs may also play a potential role in the growth and function of the placenta of pigs [36]. Interestingly, found that miR-26a was significantly up-regulated in pregnant and non-pregnant cows at 16 days post mating, and was also used as a potential biomarker in early bovine pregnancy [37]. A study identified a feasible miRNA, bta-miR 140, as an early biomarker for pregnancy diagnosis in high producing dairy cows [38]. Similarly, Lime et al proved the results of circulating bta-miR-221 and bta-miR-320a were notably expressed at over 8 weeks of pregnancy [39]. Serums from pregnant and non-pregnant mares were collected on days 12, 14, 16, and 18, and endometrial biopsies revealed 12 temporally differentially expressed miRNAs, and maternal pregnancy recognition was used as a biomarker [40]. Pathway analysis predicted that differentially expressed maternal serum exosomal miRNAs target cell growth and proliferation, as well as organ development [41], while umbilical serum exosomes and placental miRNAs may target cell development and embryonic development [42]. These studies suggest that miRNAs may play an important role in mediating the occurrence of diseases and the diagnosis of pregnancy.

Our study conducted high throughput sequencing analysis of exosomal miRNAs in pregnant and non-pregnant sows, we identified 55 miRNAs that were significantly up-regulated and 67 were down-regulated in the pregnant group compared with the non-pregnant controls, of which seven miRNAs were up-regulated more than 2-fold and 5 miRNAs were down-regulated more than 2-fold. In addition, 10 miRNAs expression levels were confirmed by RT-qPCR in this study and were found to be consistent with the high-throughput sequencing results. This confirms that the abundance of miR-192 was significantly higher in the pregnant group than in the non-pregnant control group. Therefore, these data can help us better understand the regulatory mechanisms of miRNAs, enrich the miRNA database, and ultimately help us discover novel biomarkers. High-throughput sequencing and qRT-PCR revealed that the up-regulation of miR-192 in fibrotic kidney was closely associated to the activation of the TGF-β/Smad signaling pathway. In a rat nephrectomy model, overexpression of Smad7 blocked TGF-β/Smad signaling, and thereby inhibited miR-192 expression and renal fibrosis, suggesting that miR-192 may be an important downstream of the TGF-β/Smad3 signaling pathway [43]. Adenylate cyclase7 (AC7) affects the intracellular Cyclic AMP concentrations and the differentiation of APL cells induced by all-trans-retinoic acid (ATRA). Secondly, He et al [44] found that miR-192 directly targets AC7 expression, and the knockdown of miR-192 can promote the differentiation of APL cells induced by ATRA by regulating AC7 expression. miRNA sequencing of Sika Deer revealed that miR-192 targeting MYH7 plays an important role in muscle growth and development [45]. Liang et al [46] reported that miR-192-5p maintained cell polarity through stabilizing adhesion junction protein E-cadherin during embryonic implantation, thus affecting epithelial-mesenchymal transformation.

In this study, we identified miR-192 as a candidate screening biomarker for early pregnancy given its significantly greater expression in serum exosomes of pregnant sows than in control sows. In addition, in the ROC curve analysis, the AUC of miR-192 was 0.843, indicating that exosomes miR-192 can accurately separate pregnant sows from non-pregnant controls (sensitivity of 81.0%, specific 100%). Together, this data illustrates the importance of miR-192 in the development of organisms, yet there are few studies on miR-192 in the early pregnancy of sows, and further functional studies are needed to understand their regulatory mechanisms.

The *ITGA4* gene encodes a member of the integrin chain protein family. Integrin is a heterodimeric membrane protein composed of α chain and β chain, which plays a role in cell surface adhesion and signal transduction [47]. ITGA4 is involved in the growth of blastocyst trophoblast cells, and it may be involved in the recognition and adhesion of the blastocyst and endometrium in humans [48]. In addition, the intra-uterine blockade of α4 integrin by specific antibodies resulted in failure of normal implantation, and delayed implantation in mice [49]. Furthermore, most of the regulators of the integrin family play important roles in regulating trophoblast cell function and placental preeclampsia, and ITGA4 plays an important role in regulating blastocyst invasion, trophoblast cell growth, and embryo attachment [50]. Chen et al [51] showed that miR-192 could promote the proliferation and cell cycle of prostate cancer (PCa) cells. Mao et al [52] found that miR-192 can regulate the TGF-β1/Smad signaling pathway, reduce renal fibrosis, and that miR-192 overexpression inhibits the proliferation and activation of proliferating mesangial cells. At present, many studies have shown that miRNA plays an important role in regulating cell proliferation. Garcia-Riart et al [53] found that miR-92 in chicken embryos is an important positive regulator of cell proliferation through sequencing. MiR-30s Family Inhibit the Proliferation and Apoptosis in Human Coronary Artery Endothelial Cells [47]. Woo et al [54] revealed that miR-192 inhibits the proliferation, migration, and invasion of OS cells, as well as promoting apoptosis by targeting matrix metalloproteinase-11. After that, we further studied the effect of miR-192 on BeWo cell proliferation, and the results showed that the proliferation rate slowed down after transfection. miR-192 therefore plays an important role in regulating the occurrence of diseases, growth, inflammation, and other physiological and pathological processes. ITGA4 is involved in the recognition and adhesion of blastocyst and human endometrium [48]. Our results show that miR-192 functions as early pregnancy by inhibiting ITGA4 expression. It is speculated that the two binding modes of miR-192 and the target gene may act on the target gene together. The expression of the miR-192 mimic was significantly lower than that of mir-192 mimic NC, and the expression of miR-192 inhibitor was significantly higher than that of miR-192 inhibitor NC. Therefore, we can infer that miR-192 mediates the proliferation of placental chorionic trophoblast cells by targeting ITGA4, controls tissue degeneration, and cell death in the process of embryo implantation.

## CONCLUSION

Our study identified a total of 122 statistically significant differentially expressed miRNAs in pregnant and non-pregnant samples. The results confirmed that the expression of miR-192 was significantly different between the groups (p<0.01) and can be used as a biomarker at early pregnancy. Further, miR-192 impeded embryo implantation by regulating ITGA4 in the pig uterus. After miR-192 inhibitor transfection, ITGA4 expression was significantly increased, and cell proliferation was increased. Although this experiment verified that miR-192 can regulate the expression of ITGA4, each target gene of miR-192 is still unclear, and further research is needed. Therefore, the effect of miR-192 on the biological behavior of embryo implantation may be comprehensive. It remains unclear which target genes are regulated by miR-192, and further research is required.

## Figures and Tables

**Table 1:** Primer sequences used for quantitative reverse transcription-polymerase chain reaction

Primer name	Sequences (5′-3′)	Tm (°C)
ssc-miR-10b	CCCCTCCGTTACCCTGTAGAAC	60
ssc-miR-146a-5p	TTGAGAACTGAATTCCATGGGTT	60
ssc-miR-181a	GTCGGTTCAACATTCAACGCT	60
ssc-miR-182	TTTGGCAATGGTAGAACTCACACT	60
ssc-miR-206	CGTGGAATGTAAGGAAGTGTGTG	60
ssc-miR-4334-3p	CCTGTCCTCCAGGAGCTCAA	60
ssc-miR-378	ACTGGACTTGGAGTCAGAAGGC	60
novel_138	CTCGGAAAAGGATTGGCTCTG	60
ssc-miR-199a-3p	CGCACAGTAGTCTGCACATTGGTTA	60
ssc-miR-192	TCCCTGACCTATGAATTGACAGC	60
ssc-U6-F	CGCTTCGGCAGCACATATACTA	60
ssc-U6-R	ATGGAACGCTTCACGAATTTGC	60

**Table 2:** Candidate target gene amplification primer information

Target name	Sequences (5′→3′)	Products length (bp)
*ITGA4-WT-F*	***CCCTCGAGGGA***TATTTAGGTCATCTCTGAGCTGTG	400
*ITGA4-Mut-F*	***CCCTCGAGGGA***TATTTATTCCATCTCTGAGCTGTG	
*ITGA4-WT-R*	***ATTTGCGGCCGCTTTA***TGTTTTAAACAGATATTTTGCTAAG	

**Table 3:** Real-time polymerase chain reaction primers

Target name	Sequences (5′→3′)	Products length (bp)	Tm (°C)
*ITGA4*	F: ACAGAACTGAGTAAAAGAATAGC	244	59
	R: CCGAGTATCCTAAATAACTTCCA		
*GAPDH*	F:AACATCATCCCTGCTTCTACCG	126	59
	R:GGTCAGATCCACAACCGACAC		

*ITGA4*, integrin alpha 4; *GAPDH*, glyceraldehyde-3-phosphate dehydrogenase.

**Table 4:** Kyoto encyclopedia of genes and genomes pathway analysis of candidate target genes (Partial)

Number	Kyoto encylopaedia of genes and genomes	ID	Number of genes	p-value
1	Chagas disease (American trypanosomiasis)	ssc05142	87	0.023235
2	Malaria	ssc05144	46	0.048581
3	Tuberculosis	ssc05152	127	0.049525
4	Phagosome	ssc04145	109	0.050768
5	TNF signaling pathway	ssc04668	83	0.056622
6	Rheumatoid arthritis	ssc05323	68	0.058168
7	Pertussis	ssc05133	58	0.063706
8	Phosphatidylinositol signaling system	ssc04070	62	0.064937
9	Chronic myeloid leukemia	ssc05220	55	0.067256
10	NF-kappa B signaling pathway	ssc04064	69	0.07069
11	Arginine and proline metabolism	ssc00330	45	0.072669
12	Acute myeloid leukemia	ssc05221	44	0.077929
13	B cell receptor signaling pathway	ssc04662	56	0.083151
14	Inflammatory bowel disease (IBD)	ssc05321	49	0.08672
15	T cell receptor signaling pathway	ssc04660	79	0.089109
16	Pancreatic cancer	ssc05212	49	0.099677
17	Production	ssc04672	36	0.100078
18	Thyroid hormone signaling pathway	ssc04919	82	0.103552
19	FoxO signaling pathway	ssc04068	95	0.104459
20	Toll-like receptor signaling pathway	ssc04620	74	0.105498
21	Legionellosis	ssc05134	49	0.11387
22	Hepatitis B	ssc05161	106	0.11436
23	Carbon metabolism	ssc01200	72	0.117704
24	Epstein-Barr virus infection	ssc05169	130	0.120144
25	Insulin signaling pathway	ssc04910	94	0.120151
26	Fc gamma R-mediated phagocytosis	ssc04666	60	0.120672
27	Inositol phosphate metabolism	ssc00562	44	0.120712
28	mTOR signaling pathway	ssc04150	46	0.121107
